# Systematic analysis of factors affecting the efficacy of corneal reinnervation surgery in treating neurotrophic keratitis

**DOI:** 10.1186/s40001-025-02992-8

**Published:** 2025-08-13

**Authors:** Rui Kuang, Shuqia Xu, Xunxun Lin, Bo He, Yangbin Xu, Zhaowei Zhu

**Affiliations:** 1 Department of Plastic Surgery, The First Affiliated Hospital of Sun Yat-Sen University, No. 58 Zhongshan Road 2, Guangzhou, 510080 Guangdong China; 2 Orthopaedic Trauma and Joint Department, Department of Orthopaedics, The Third Affiliated Hospital of Sun Yat-Sen University, Guangzhou, 510000 China

**Keywords:** Corneal neurotization, Neurotrophic keratitis, Corneal sensation, Corneal nerve transplantation

## Abstract

**Objective:**

This study investigated the efficacy of corneal neurotization (CN) surgery for treating neurotrophic keratitis (NK) and evaluated potential factors affecting treatment outcomes.

**Methods:**

Literature databases were searched from the inception to January 2024 for retrospective or prospective studies related to corneal neurotization for NK treatment. Data analysis was performed using SPSS 26.0, including analysis of variance, rank sum tests, and multiple regression analysis to determine the efficacy of CN and the impact of patients’ age, gender, surgical method, and etiology on surgical outcomes.

**Results:**

A total of 14 retrospective or prospective studies were included, comprising 191 patients who underwent CN for neurotrophic corneal lesions. CN significantly improved Mackie staging, logMAR visual acuity, and corneal sensation in patients with congenital and acquired NK (*p* < 0.0001). In young patients (≤ 30 years), improvement in central corneal sensation was more pronounced (*p* < 0.05), while elder patients (> 30 years) showed more significant visual acuity improvement (*p* < 0.05). Multiple linear regression analysis also showed that age was associated with the postoperative improvement in logMAR visual acuity: the elder the patients, the more improvement in visual acuity (*p* < 0.05). Compared to direct corneal neurotization (DCN), indirect corneal neurotization (ICN) showed more significant improvement in central corneal sensation (*p* < 0.01). And the patients with congenital NK got more improvement of central corneal sensation after CN by comparison with the others with acquired etiologies (*p* < 0.001). Multiple linear regression analysis suggested that congenital etiology was associated with more significant postoperative improvement in corneal sensation (*p* < 0.05).

**Conclusion:**

CN surgery significantly improves visual acuity, NK Mackie staging, and corneal sensation in NK patients. Age, etiology, and surgical technique may significantly influence treatment outcomes.

**Supplementary Information:**

The online version contains supplementary material available at 10.1186/s40001-025-02992-8.

## Introduction

Neurotrophic keratitis (NK) is a rare degenerative ocular disorder caused by trigeminal nerve damage, with an incidence of approximately 5/10,000 [1–3]. The cornea contains neural network, making it one of the most densely innervated and sensitive parts of the human body [4]. This network includes the limbal nerve plexus, stromal nerve plexus, subepithelial nerve plexus beneath Bowman’s membrane, subbasal nerve plexus above Bowman’s membrane, and intraepithelial nerve terminals, primarily originating from the ophthalmic branch of the trigeminal nerve’s sensory nerves, which mediating responses to pain, cold, heat, chemical, and mechanical stimuli [4–7]. Healthy nerves also provide important trophic support to corneal epithelial cells by releasing various neurotrophic factors to maintain corneal epithelial integrity [8, 9]. Additionally, corneal nerves regulate corneal epithelial renewal by modulating activity of limbal stem cells (LSCs) in the basal epithelium of the corneal limbus [10–12].

Corneal denervation may arise from diverse etiologies affecting any level of the neural pathway, from central nuclei to peripheral nerve endings [8]. The most common etiologies include viral herpetic infections, chemical burns, and surgical trauma [13]. Other causes include intracranial tumors, long-term use of corneal contact lenses, keratoconus surgery, diabetes, dry eye syndrome, and familial dysautonomia [2].

Corneal nerve injury leads to reduced or absent corneal sensation, epithelial defects, and impaired corneal healing [8], ultimately resulting in corneal damage, decreased vision, and potentially permanent blindness [14]. Due to reduced corneal sensation, early NK symptoms are often subtle, with most patients initially experiencing only eye redness, photophobia, tearing, decreased vision, and eye fatigue [15, 16], which may lead to delays in diagnosis and treatment.

In 2009, Terzis et al. first described corneal neurotization (CN) surgery using contralateral supratrochlear and supraorbital nerves [17], which provided a fundamental treatment for NK by transplanting healthy nerves to denervated corneas. This approach yielded promising results. Subsequently, CN techniques have been continuously updated and improved, leading to the development of two main surgical approaches: direct corneal neurotization (DCN), which directly transfers healthy nerve branches to the affected cornea, and indirect corneal neurotization (ICN), which uses autologous or allogeneic nerve grafts [18]. The choice of technique depends on factors such as the availability of donor sensory nerves, distance from the affected cornea, and surgeon’s experience and preference [19], but the superiority of outcomes between techniques remains controversial.

Therefore, this study aims to systematically review the postoperative efficacy of CN cases, evaluate the effectiveness of CN in treating NK, and identify potential factors influencing surgical outcomes, providing reference for establishing CN surgical indications and making appropriate clinical treatment decisions.

## Methods

Prior to conducting this systematic review, a detailed study protocol (including review questions, search strategy, and inclusion/exclusion criteria) was established. The review followed PRISMA 2020 Statement standards [20] to evaluate both the efficacy of corneal neurotization (CN) surgery and factors influencing clinical outcomes.

### Search strategy

Databases including China Biology Medicine disc (CBM, http://www.sinomed.ac.cn/), Pubmed (https://pubmed.ncbi.nlm.nih.gov/), Embase (https://www.embase.com/), the Cochrane Library (https://www.cochranepubliclibrary.ca/), Web of Science (https://access.clarivate.com) were searched for all studies related to CN treatment for NK from database inception to January 2024, without language restrictions. Search terms included “corneal neurotisation,” “corneal neurotization,” “corneal reinnervation,” “corneal nerve regeneration,” “corneal nerve transfer,” and “corneal nerve grafting.”

### Inclusion and exclusion criteria

Inclusion criteria:

1. Study subjects: Patients diagnosed with NK.
2. Intervention: Patients receiving various corneal nerve transplantation surgeries.
3. Study type: Retrospective and prospective studies. Currently, there is a lack of randomized controlled trials (RCTs) on corneal neurotization in the treatment of neurotrophic keratitis.
4. Study outcomes: NK Mackie staging, corneal sensation, visual acuity, and surgical complications.

Exclusion criteria:

1. Animal experiments, cell studies, reviews, meta-analyses, conference abstracts, case reports, or letters.
2. Studies with fewer than 3 patients.
3. Low-quality literature with missing study data, design flaws, or unscientific data analysis methods.

### Literature screening and data extraction

Literature screening: (1) All retrieved literature was imported into EndNote, with duplicate articles removed by two independent reviewers; (2) Titles and abstracts were read to further eliminate duplicates and irrelevant literature. Initial screening was conducted based on inclusion criteria, and full-text review was performed for final inclusion; (3) Data extraction and analysis were conducted for included studies.

Data extraction: The following data were extracted according to the data extraction form: General study information: authors, publication year, and sample size; Clinical characteristics: age, gender, NK etiology, age at denervation, duration of denervation, and follow-up time; Interventions: CN surgical method; Clinical outcomes: (1) best-corrected visual acuity, (2) NK Mackie staging, (3) central corneal sensation, and (4) complications at donor and recipient sites.

### Literature quality assessment

Two reviewers familiar with the Methodological Index for Non-Randomized Studies (MINORS) independently assessed each included study. Disagreements were resolved through discussion or consultation with a third reviewer if necessary. MINORS consists of 12 items, each scored 0–2 (0 indicating unreported, 1 indicating reported but inadequate information, 2 indicating reported with adequate information). The first 8 items apply to non-comparative studies with a maximum score of 16; all 12 items apply to comparative studies with a maximum score of 24. Methodological quality assessment of this systematic review was performed by 2 researchers according to the Systematic Review Tool 2 (AMSTAR 2) [21].

### Statistical analysis

Corneal sensation quantified using standard Cochet–Bonnet esthesiometry was collected in millimeters (mm) to analyze changes in central corneal sensation after CN. All twelve studies evaluating corneal sensation changes following neurotization surgery exclusively utilized Cochet–Bonnet esthesiometry for pre- and postoperative assessments. NK Mackie staging data were collected. For statistical analysis convenience, Snellen and decimal visual acuities reported in the literature were converted to logarithm of the minimum angle of resolution (LogMAR) visual acuity. Lower LogMAR values indicate better visual acuity. Counting fingers and hand motion vision were converted as follows: counting fingers 1.9 logMAR, hand motion 2.3 logMAR. Appropriate analysis of variance, Mann–Whitney *U* test, or Student’s *t*-test was performed for categorical and continuous variables. Multiple linear regression analysis was conducted to evaluate the factors affecting the postoperative central corneal sensation and visual acuity.

Except for visual acuity values conforming to normal distribution and expressed as mean ± standard deviation, other indicators not conforming to normal distribution were calculated using the quartile method. The significance level was set at *α* < 0.05. Data analysis and graphics were performed using SPSS software (version 26; IBM SPSS Statistics, Armonk, NY) and GraphPad Prism  (version 9.5; GraphPad Software, Inc., San Diego, California).

## Results

### Literature screening results and basic characteristics of included studies

**Fig. 1:**
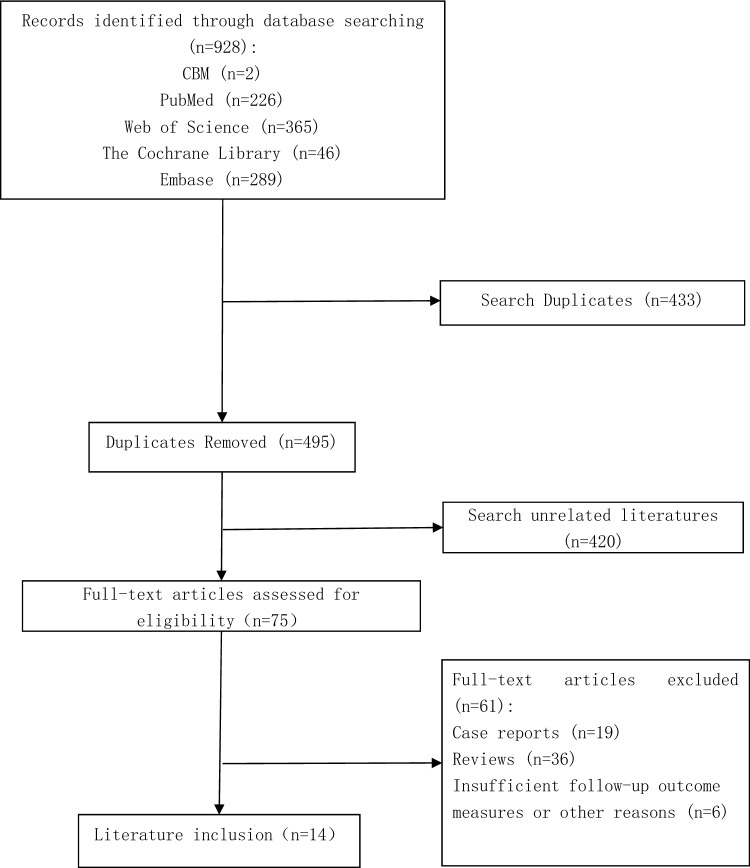
PRISMA flow diagram of literature search and selection process for the systematic review on the efficacy of corneal neurotization in treating neurotrophic keratitis

**Fig. 2:**
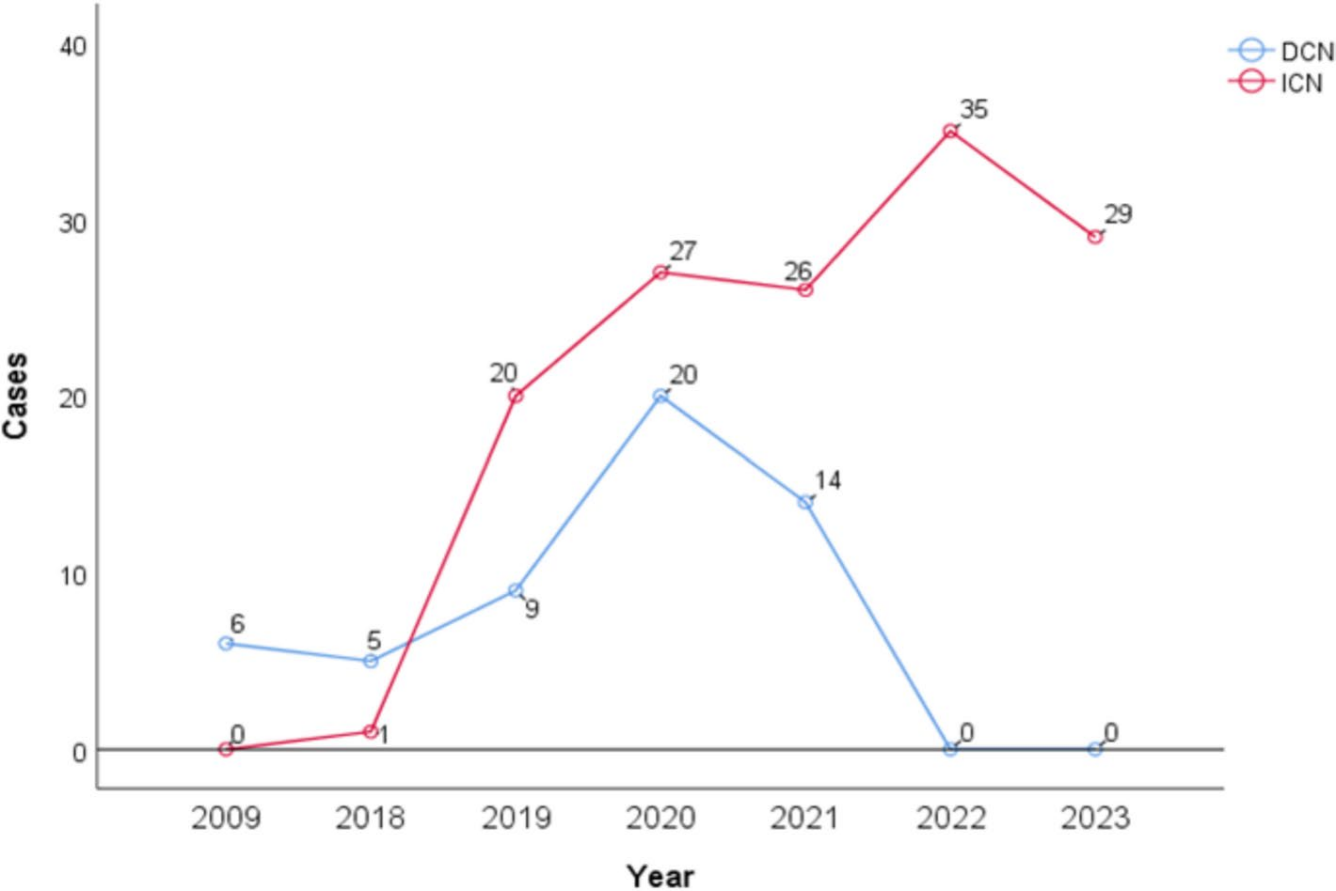
Line chart showing the number of published corneal neurotization cases each year. Blue represents the number of direct corneal neurotization cases and red represents the number of indirect corneal neurotization cases

Based on the literature search strategy, 14 English-language articles were ultimately included. The specific screening process is shown in Fig. 1. The included studies were categorized and summarized (Table 1), revealing that indirect corneal neurotization surgery was predominant in these studies (Fig. 2). The Methodological Index for Non-Randomized Studies (MINORS) [22] was used to assess the quality of included literature, with all studies scoring 9 points or higher, indicating reliable research quality. MINORS scoring criteria and specific scores for included studies are shown in Supplementary Tables 1 and 2.

**Table 1:** Abstract of included studies

Authors/Years	Patients/eyes	Sex	Age(y)	Etiology	Duration of Denervation (mo)	Follow-up time (mo)	MINORS scores
		F/M		Congenital/Acquired			
Su et al. [23]	18/18	3/15	34.83 ± 9.85	0/18	44.20 ± 60.04	24	10
Saini et al. [24]	11/11	7/4	44.55 ± 21.38	0/11	31.68 ± 8.04	10.09 ± 2.43	13
Wu et al. [25]	12/12	2/10	32.5 ± 8.5	0/12	44.4 ± 60.0	24.7 ± 7.1	12
Woo et al. [26]	23/28	11/12	15.6 ± 13.6	14/9	77.4 ± 60.8*	37.8 ± 22.5	11
Rafailov et al. [27]	23/24	13/10	60.2	2/21	28.5*	12.2	9
Kim et al. [28]	6/6	4/2	77.9	0/6	11.8	11.3	9
Elalfy et al. [29]	11/11	3/8	43	0/11	–	14.5	11
Wisely et al. [30]	4/5	2/2	70.5	0/4	17.8	15.8	9
Sweeney et al. [31]	17/17	5/12	42.6(4–69)	2/15	–	–	9
Fogagnolo et al. [18]	25/26	5/20	45.44	1/24	57.44 ± 67.78	–	15
Lin et al. [32]	13/13	7/6	61.8	0/13	182.4	18.5	12
Catapano et al. [33]	16/19	7/9	12.5 ± 8.3	11/5	76.8 ± 75.6	–	11
Weis et al. [34]	6/6	5/1	57	0/6	23	12	11
Terzis et al. [17]	6/6	2/4	37	0/6	17.8	16.3 ± 2.42	10

77.4 ± 60.8 * indicates the mean ± standard deviation of denervation duration in the 8 patients with NK due to surgical trauma in this study

28.5 * indicates the median denervation duration of 22 patients documented in this study

– indicates the data were not recorded

### Comparison of basic information

This study included 191 patients (202 eyes, Table 2) who underwent corneal nerve transplantation, with a mean age of 41 years, including 76 males (39.8%) and 115 females (60.2%). The median duration of corneal denervation was 30 months, and the median postoperative follow-up duration was 14 months. Among the patients included in the analysis, 28 (14.66%) had congenital etiologies, with trigeminal nerve hypoplasia being the most common (6 patients, 21.4%). Acquired etiologies accounted for 163 patients (85.34%), with craniocerebral tumors (69 patients, 42.3%) and corneal herpetic infections (HSV/HZV, 45 patients, 27.6%) being the most common.

**Table 2:** Data Statistics 0f Included Cases

Patients (*n*)	191
Eyes (*n*)	202
Male (*n*)	76 (39.8%)
Female (*n*)	115 (60.2%)
Age(y)	41.05 ± 23.52
Etiology-Congenital	28 (14.66%)
Trigeminal hypoplasia	6 (21.4%)
Craniocerebral hypoplasia	6 (21.4%)
Congenital corneal hypoesthesia	10 (35.7%)
Others	6 (21.4%)
Etiology-Acquired	163 (85.34%)
Craniocerebral tumor	69 (42.3%)
HSV/HZV	45 (27.6%)
Trauma	11 (6.75%)
Trigeminal nerve palsy	11 (6.75%)
Multiple eye surgeries	8 (4.9%)
Cerebrovascular malformation	7 (4.29%)
Facial paralysis	4 (2.45%)
Others	8 (4.9%)
Duration of Denervation (mo, *n* = 118)	30 (18, 52)
Follow-up time (mo, *n* = 119)	14 (10, 24)

Further analysis of data distribution for different etiologies, surgical techniques, and nerve donor site selection revealed that in the congenital etiology group, most patients received indirect corneal neurotization rather than direct neurotization (92.86% vs. 7.14%). Similarly, for acquired etiology cases, more patients received indirect corneal neurotization (71.17% vs. 28.22%). In patients undergoing direct corneal neurotization, the ipsilateral (affected side) supraorbital/supratrochlear nerve (50.0%) was most commonly used as the donor nerve due to distance limitations between donor and recipient sites. In indirect corneal neurotization, the contralateral (healthy side) nerve was more often chosen as the donor nerve (59.86% vs. 20.42%), with the contralateral supraorbital nerve being the most common donor nerve (28.87%). In the acquired etiology group, 7 patients used the ipsilateral (affected side) infraorbital nerve as the donor nerve. Regarding nerve graft selection, 17 patients with acquired etiologies used allogeneic decellularized nerves (11.97%), while the remaining patients used autologous sural nerves as grafts (88.03%) (Supplementary Table 3).

### Analysis of corneal neurotization surgery efficacy

**Fig. 3:**
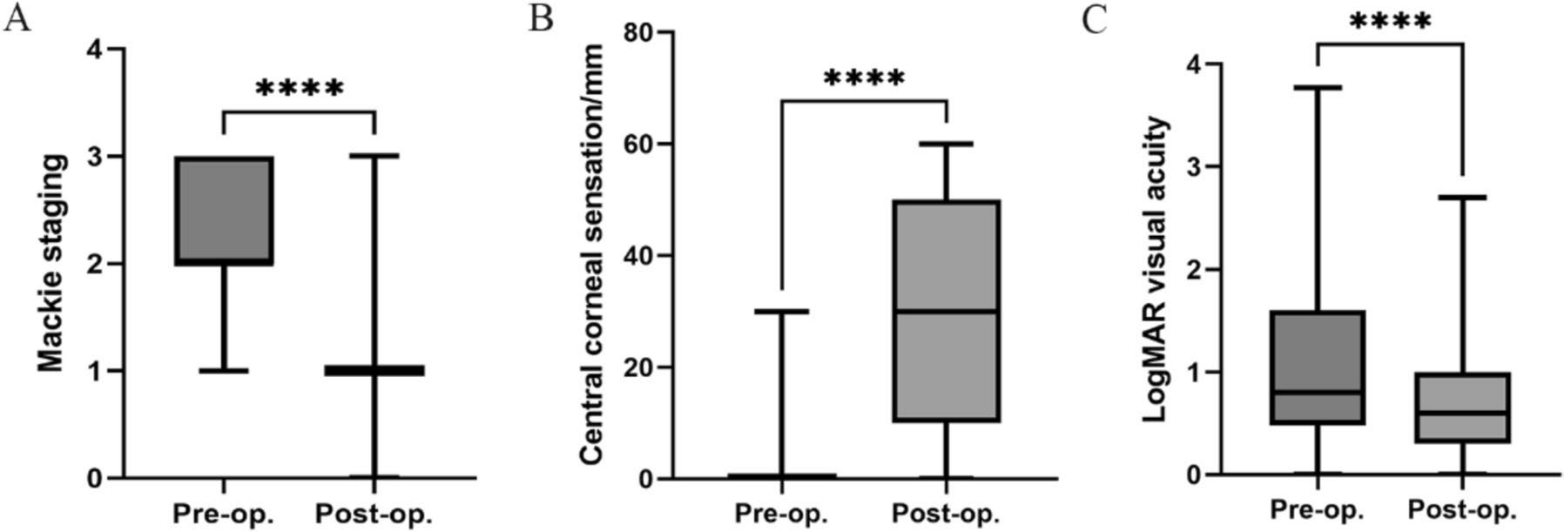
Comparison of preoperative and postoperative indicators. **A** Preoperative and Postoperative NK Mackie staging; **B** Preoperative and Postoperative central corneal sensation; **C** Preoperative and Postoperative logMAR visual acuity. ****:  *p*< 0.0001

Statistical analysis was performed on NK Mackie staging, central corneal sensation (mm), and logMAR visual acuity before and after CN. Due to non-normal distribution of data, quartile methods were used for representation and rank sum tests for statistical analysis. Results showed significant improvements in Mackie staging, central corneal sensation, and visual acuity after CN for all etiologies (*p* < 0.0001, Fig. 3).

### Analysis of factors affecting postoperative central corneal sensation

**Fig. 4:**
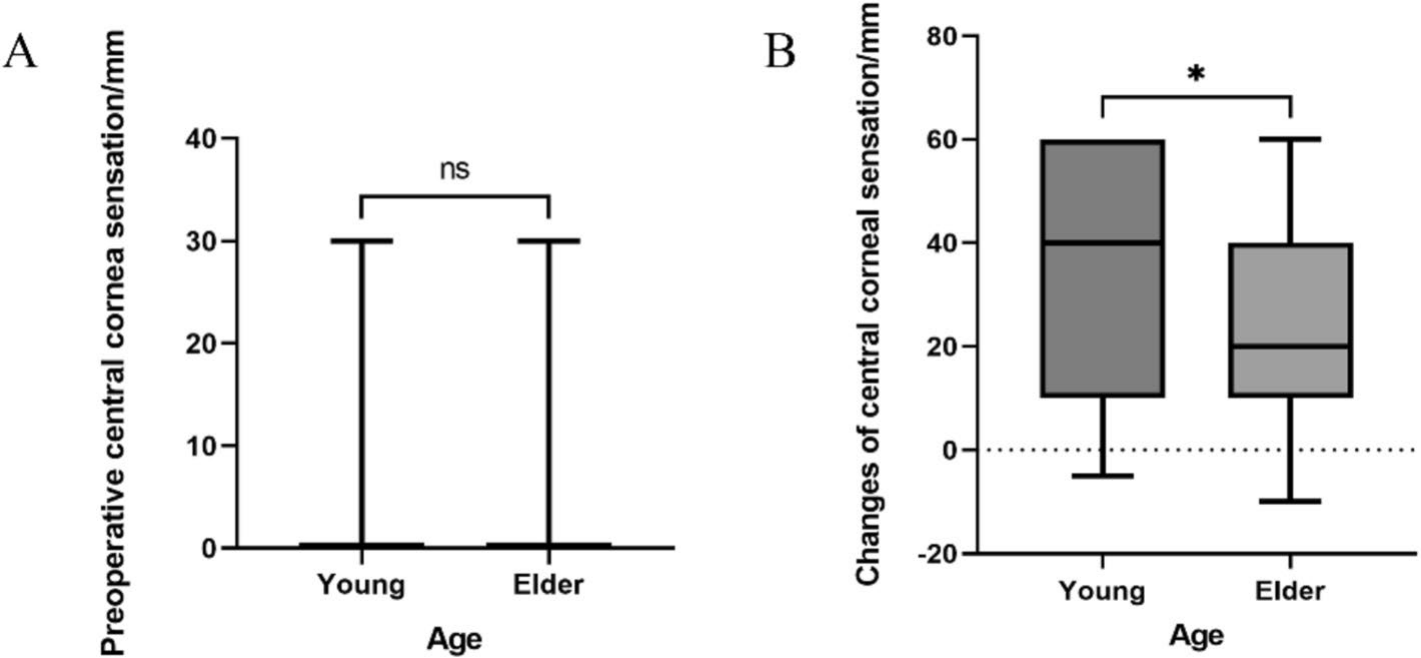
**A** Preoperative central corneal sensation in different age groups; **B** Changes of central corneal sensation in different age groups. *: *p* < 0.05

Patients were stratified by age into young group (≤ 30 years) and elder group (> 30 years). Rank sum test results showed no significant statistical difference in preoperative central corneal sensation between the two age groups (*p* > 0.05, Fig. 4A). Younger patients showed significantly greater improvement in central corneal sensation compared to elder patients (*p* < 0.05, Fig. 4B).

**Fig. 5:**
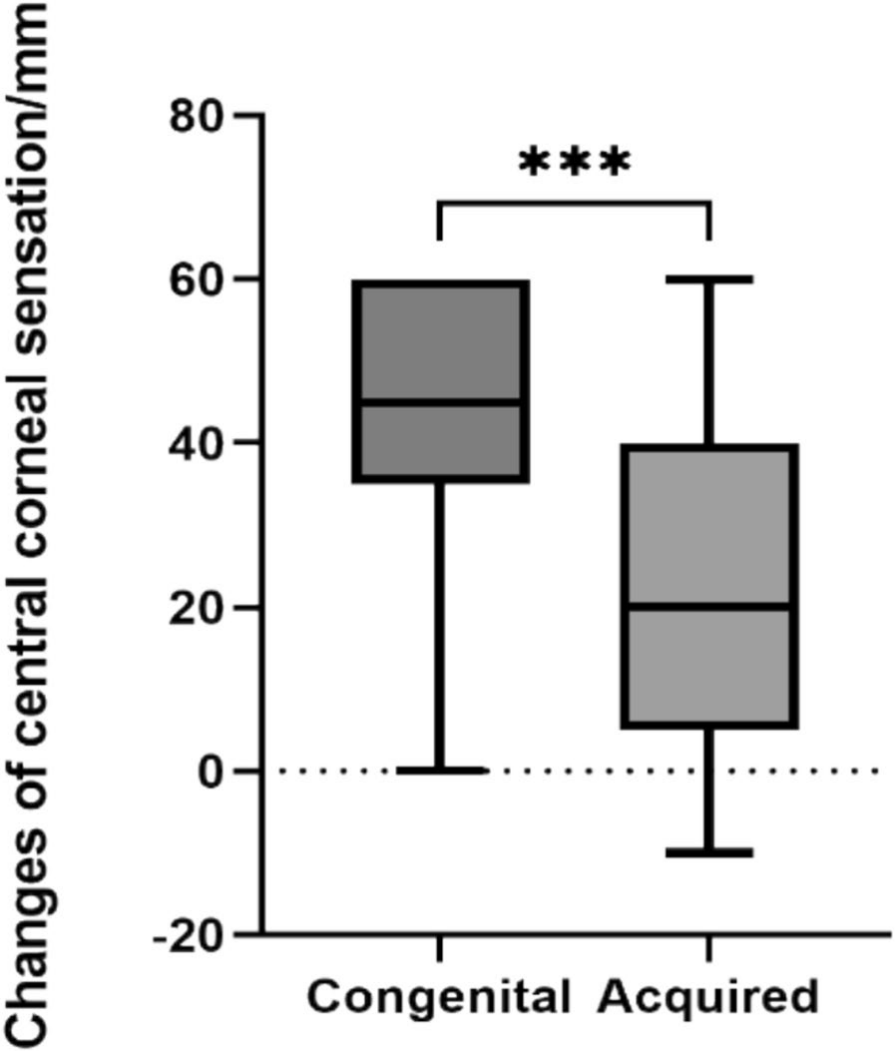
The improvement of central corneal sensation in patients with different etiologies. ***: *p* < 0.001

Additionally, patients with congenital etiologies showed better improvement in central corneal sensation compared to those with acquired etiologies (*p* < 0.001, Fig. 5).

**Fig. 6:**
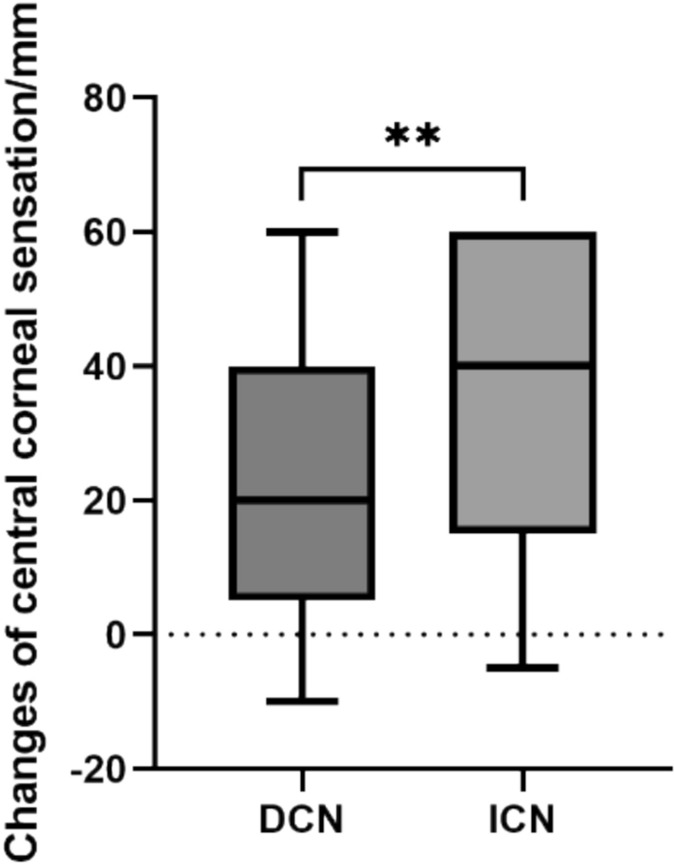
The improvement of central corneal sensation after different surgeries. **: *p* < 0.01

Patients who underwent ICN surgery showed better recovery of central corneal sensation (*p* < 0.01, Fig. 6).

Multiple linear regression analysis of the correlation between age, etiology, and surgical technique with postoperative changes in central corneal sensation revealed a linear correlation between etiology and degree of central corneal sensation improvement (*p* < 0.05, Table 3), with congenital etiology patients achieving more significant improvement in central corneal sensation postoperatively. However, no significant correlations were found between age, surgical technique, and sensation improvement (*p* > 0.05, Table 3).

**Table 3:** Multiple linear regression analysis of central corneal sensation

	*B*	*β*	*t*	*P*	*F*	Adjusted R2
Age	− 0.183	− 0.193	− 1.680	0.096	8.089	0.183
Etiology	14.679	0.260	2.404	0.018*		
Surgical Method	− 6.731	− 0.125	− 1.227	0.223		

*p < 0.05

### Analysis of factors affecting postoperative visual acuity

**Fig. 7:**
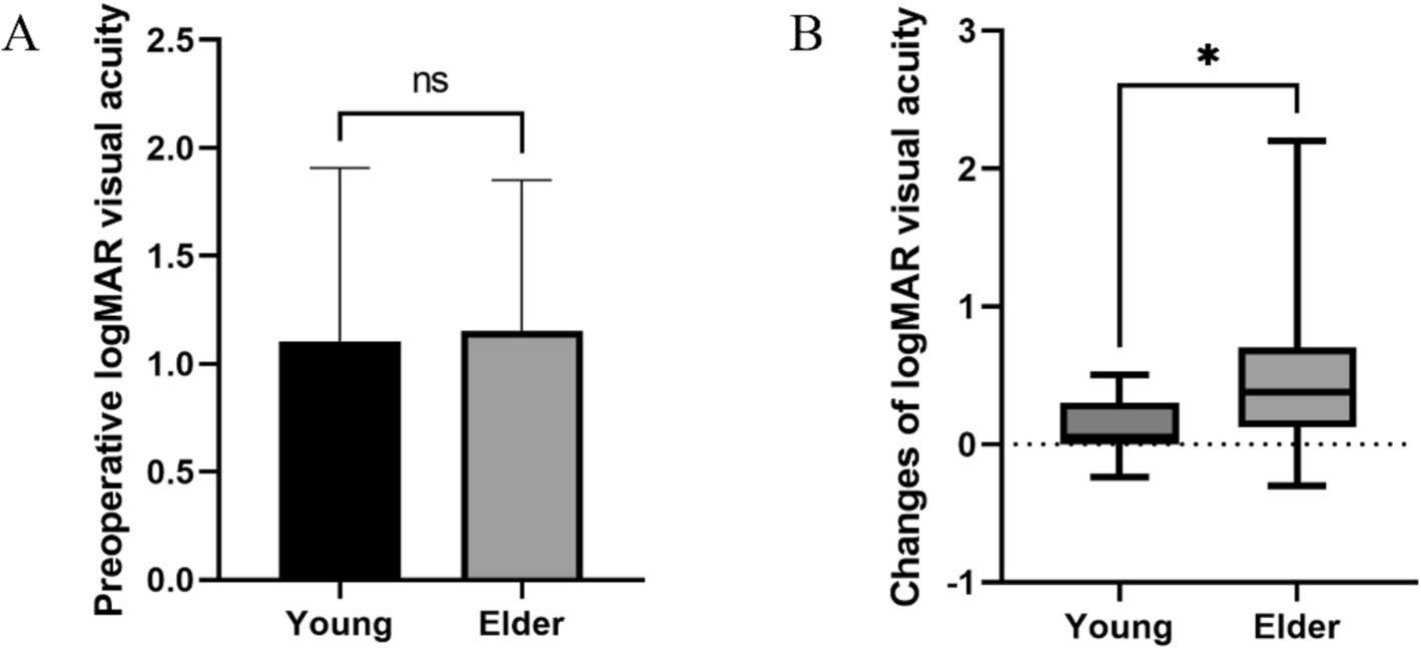
**A** Preoperative logMAR visual acuity in different age groups; **B** Changes of logMAR visual acuity in different age groups. *: *p* < 0.05

*T*-test results showed no significant statistical difference in preoperative visual acuity between different age groups (*p* > 0.05, Fig. 7A). Elder patients showed more significant visual acuity improvement after CN surgery (*p* < 0.05, Fig. 7B).

Multiple linear regression analysis of the impact of age, gender, and surgical technique on postoperative logMAR visual acuity changes showed a positive linear correlation between age and visual acuity improvement (*p* < 0.05, Table 4), with elder patients achieving more significant visual acuity improvement. However, no significant correlations were found between patient gender, surgical technique, and degree of visual acuity improvement (*p* > 0.05, Table 4).

**Table 4:** Multiple linear regression analysis of logMAR visual acuity

	*B*	*β*	*t*	*P*	*F*	Adjusted R2
Age	0.14	0.395	2.475	0.018*	3.633	0.176
Sex	0.155	0.107	0.689	0.495		
Surgical Method	0.317	0.179	1.091	0.283		

*p < 0.05

### Summary of surgical complications

Analysis of postoperative complications recorded in the 14 included studies revealed that 11 studies reported adverse events, primarily sensory abnormalities and pain in the facial donor area. Other reported adverse events included immediate postoperative mild facial edema [18], exposure of ocular sutures [33], and subconjunctival neuroma [17], with low incidence rates. According to statistics, 54 patients (28.27%) experienced temporary numbness in the frontal nerve donor area postoperatively. 25 patients (13.09%) developed immediate postoperative facial edema. Other recorded complications such as pain in donor site, itching, bone spur formation, suture exposure in recipient site, and subconjunctival neuroma occurred at low rates (Table 5).

**Table 5:** Summary of Surgical Complications

	Complication	Patients
Donor site complications	Numbness	54
	Pain	3
	Itching	3
	Bone spur	1
Recipient complications	Facial edema	25
	Suture exposure	4
	Subconjunctival neuroma	1

## Discussion

Our systematic analysis of 14 studies on corneal neurotization (CN) surgery for neurotrophic keratitis (NK) confirms the intervention’s efficacy in restoring corneal sensation and identifies key factors influencing clinical outcomes. These findings align with prior reports demonstrating that the majority of patients included in the analysis had acquired NK, commonly secondary to intracranial tumors (42.3%) and corneal herpetic infections (27.6%), while congenital NK patients were less common, primarily due to trigeminal nerve hypoplasia (21.4%).

The treatment of NK relies on early diagnosis and timely intervention, with the ultimate goal of restoring corneal nerve sensation and trophic function. Mackie staging is an important basis for determining treatment strategies. Proposed by Mackie IN in 1995, it is a staging system used to assess the degree of corneal damage in neurotrophic keratitis [35]. According to Mackie classification, NK can be divided into three stages: mild (stage 1), moderate (stage 2), and severe (stage 3) [36]. Stage 1 represents the initial phase of corneal damage, with intact corneal epithelium but accompanied by punctate keratopathy; Stage 2 is characterized by persistent corneal epithelial defects; at Stage 3, persistent corneal epithelial defects lead to stromal tissue melting or even perforation. At Mackie stage 3, refractory corneal ulcers cannot be alleviated through medical treatment and require surgical intervention, such as amniotic membrane transplantation, tarsorrhaphy, and conjunctival flap surgery, to reduce corneal exposure and promote healing [8, 37].

In 1972, Samii [38] first proposed the concept of CN, attempting to connect the greater occipital nerve to the proximal optic nerve using sural nerve grafts. But the results were unsatisfactory and the surgery was highly traumatic, leading to no further research or application. It was not until 2009 that Terzis [17] described a less traumatic and more effective CN technique using healthy supraorbital and supratrochlear nerves as donor nerves for direct CN to treat NK. Since then, CN has been continuously practiced and improved, with more surgical techniques being proposed. Minimally invasive direct CN (MIDCN) and minimally invasive indirect CN (MICN) were introduced and applied, further reducing complications associated with large incision surgical methods. The efficacy of CN has been proven to be reliable and stable [18, 30, 31, 33, 39–41]. As a surgical treatment targeting the root cause of NK, CN can restore corneal nerve sensation and trophic function, improve ocular surface homeostasis, and address the fundamental cause of NK—corneal nerve damage—providing a new direction for treating refractory NK. This surgery can maintain corneal integrity by restoring corneal sensation and its trophic support, promoting epithelial wound healing, and inhibiting stromal melting [42]. In vivo confocal microscopy has also shown an increase in corneal nerve length and density after CN [18, 25, 29, 39, 43, 44]. Currently, CN has been applied in both adults and children with certain efficacy [25, 45].

For efficacy evaluation of NK treatment, indicators such as corneal sensation, best-corrected visual acuity, extent of corneal epithelial defects, and corneal nerve diameter, density, and length are commonly used [23–25]. Other indicators include tear secretion tests, tear film break-up time for ocular surface quality assessment [24, 29], and patients’ subjective feelings about ocular sensitivity [27, 30]. Literature summaries show that corneal sensation can significantly improve after CN [19]. However, postoperative visual acuity improvement is limited by factors such as corneal scarring, amblyopia, and other potential ocular diseases [33, 46–48]. Regardless of the surgical technique, CN can significantly promote corneal epithelial defect repair and improve Mackie staging [18, 49]. Notably, the two included studies evaluating both corneal nerve regeneration and sensation demonstrated that morphological nerve regeneration consistently precedes functional sensory recovery [23, 24]. In contrast, ocular surface restoration correlates strongly with sensory recovery [24, 26], suggesting that corneal epithelial healing represents the most clinically significant treatment indicator in neurotrophic keratitis (NK). Consequently, proactively stabilizing the ocular surface before functional corneal recovery may optimize outcomes following neurotization surgery. This supports postoperative protective interventions (e.g., temporary tarsorrhaphy) as essential protective measures during the recovery phase [26].

We did a statistical analysis of the included patients’ preoperative and postoperative NK Mackie staging, central corneal sensation, and logMAR visual acuity. The results showed that regardless of etiology, Mackie staging, central corneal sensation, and visual acuity all improved significantly postoperatively, suggesting that CN can indeed improve symptoms and prognosis in NK patients.

CN surgical techniques include DCN [33, 50] and ICN [51]. DCN transfers the contralateral or ipsilateral supraorbital/supratrochlear nerves to the anesthetic cornea, and ICN connects supraorbital/supratrochlear nerves to the affected cornea through a nerve graft including autograft and processed nerve allograft (PNA). DCN involves greater surgical trauma, while ICN using autografts can lead to additional complications such as sensory abnormalities in the nerve donor site [52, 53]. And ICN has a longer recovery time compared to DCN. Therefore, there is currently controversy regarding the superiority of outcomes between these two surgical techniques. Fung et al. [54] proposed that DCN results in less perineural scarring and more axons compared to ICN, thus providing better outcomes. However, Swanson’s study showed that ICN could better improve corneal sensation [55]. Other studies have found no significant difference between the two surgical techniques [18]. Our research found that ICN can achieve more significant improvement in central corneal sensation compared to DCN. However, heterogeneity in baseline patient characteristics prevents definitive conclusions regarding comparative efficacy, contributing to ongoing controversy between direct corneal neurotization (DCN) and indirect corneal neurotization (ICN). The underlying mechanisms causing outcome differences remain undetermined and require further investigation. Sural nerve autografts currently represent the standard graft material for ICN. Notably, surgeons have recently introduced processed nerve allografts (PNAs) to reduce donor-site morbidity. Emerging retrospective evidence indicates that ICN using PNAs achieves comparable safety, corneal sensory recovery, and nerve regeneration rates to autografts [31, 48]. This aligns with established efficacy of PNAs in peripheral nerve reconstruction elsewhere [56]. Nevertheless, clinical experience with PNAs in ICN remains limited, highlighting the need for prospective studies to validate their efficacy and establish protocols for widespread adoption.

Through analyzing the effectiveness of CN and the factors influencing efficacy, we found that both congenital and acquired NK patients showed significant improvements in Mackie staging, central corneal sensation, and visual acuity after CN. Younger patients showed better improvement in central corneal sensation postoperatively compared to elder patients, but their visual acuity improvement was not as pronounced as in elder patients. Swanson’s study also found similar patterns [55]. We consider that this might be due to varying degrees of amblyopia in younger patients interfering with visual acuity improvement. While corneal neurotization (CN) surgery effectively restores sensation and promotes epithelial healing, final visual outcomes remain contingent on pre-existing ocular comorbidities [28]. Crucially, delayed intervention in advanced neurotrophic keratitis (NK) often results in vision-limiting stromal scarring despite epithelial closure. Therefore, early surgical intervention is strongly recommended to maximize visual potential, particularly in pediatric patients where persistent corneal opacity risks irreversible amblyopia [27, 28]. Subsequent corneal transplantation can address established scarring, with documented cases achieving successful visual rehabilitation after CN surgery. Notably, all reported cases maintained normal re-epithelialization post-transplant [33]. A critical determinant of sensory recovery is denervation duration, with prolonged denervation correlating significantly with slower functional recovery [26]. This phenomenon likely reflects chronic denervation pathophysiology, wherein Schwann cell dysfunction, basal lamina alterations, and upregulated inhibitory molecules impede axonal regeneration [57]. Consistent with our statistical results, pediatric patients demonstrate superior sensory recovery outcomes after CN [27], potentially attributable to enhanced neuroregenerative capacity in younger individuals [46, 58]. In this study, we have not observed any apparent correlation between gender and postoperative outcomes.

Regarding postoperative complications, ICN patients may experience complications related to sural nerve deficiency at the donor site, such as sensory loss, discomfort, and abnormal pain in the calf or foot [59]. However, these complications become less noticeable 3 months after surgery. Ducic’s study analyzed chronic complications and donor site morbidity after autologous sural nerve grafting, finding that 22.9% of patients may experience chronic pain, 7% may have wound complications, and 7.9% may have symptoms significantly affecting daily life [60]. Catapano et al. found that although the sural nerve donor site lost innervation, most patients had low morbidity, with half of the patients subjectively assessing no sensory loss, a quarter occasionally experiencing pain, numbness, or cold hypersensitivity, and one patient reporting inability to perform activities due to foot pain sensation [61]. Other studies have reported the impact of surgery on frontal nerve donor site sensation, finding that postoperative numbness in the frontal nerve donor area was only temporary. The patients reported frontal numbness for 3–6 months postoperatively, but this numbness quickly disappeared, and frontal sensation returned to preoperative levels at about 12–18 months [18, 25, 32]. In Wisely’s study, all patients’ subjective skin sensation in the nerve donor area returned to normal [30]. In Terzis’s study [17], three patients reported frontal itching and pain immediately after surgery, but without eye pain, and recovered to normal within a few months. Combined with the relevant studies included in this research, we found that the most common complication of CN for neurotrophic keratitis is temporary, acceptable sensory abnormalities due to denervation, which are usually tolerable for patients, and most patients expressed satisfaction with the surgical results of CN.

### Study limitations and prospects

This study still has limitations. On one hand, it lacks randomized controlled trials (RCTs), and the indicators for efficacy evaluation used in various studies are not entirely uniform. Follow-up times, data quality, level of detail, and available preoperative patient data vary greatly across studies. Ideally, strict case selection criteria should be applied, randomized controlled studies should be conducted, and close follow-up should be performed to assess the impact of factors such as patient age, etiology, duration of denervation, and surgical technique on surgical outcomes. On the other hand, the principles of corneal nerve regeneration after nerve transplantation are not yet fully clear, requiring more rigorous studies with more cases for verification.

## Conclusion

CN surgery can significantly improve visual acuity, NK Mackie staging, and corneal sensation in NK patients. Age, etiology, and surgical technique may significantly influence treatment outcomes.

## Supplementary Information

Additional file 1.

## Data Availability

No datasets were generated or analysed during the current study.
